# On the role of the proventricle region in reproduction and regeneration in *Typosyllis antoni* (Annelida: Syllidae)

**DOI:** 10.1186/s12862-016-0770-5

**Published:** 2016-10-04

**Authors:** Michael Weidhase, Patrick Beckers, Christoph Bleidorn, M. Teresa Aguado

**Affiliations:** 1 Molecular Evolution & Animal Systematics, Institute of Biology, University of Leipzig, Talstraße 33, D-04103 Leipzig, Germany; 2 Zoology and Evolutionary Biology, Institute of Evolutionary Biology and Ecology, Rheinische Friedrich-Wilhelms-Universität Bonn, An der Immenburg 1, D-53121 Bonn, Germany; 3 Museo Nacional de Ciencias Naturales, Spanish Research Council (CSIC), José Gutiérrez Abascal 2, 28006 Madrid, Spain; 4 Departamento de Biología, Facultad de Ciencias, Universidad Autónoma de Madrid, Cantoblanco, 28049 Madrid, Spain

**Keywords:** Confocal laser scanning microscopy (cLSM), Proventricle, Schizogamy, Scissiparity, Stolon

## Abstract

**Background:**

Syllids are a species rich annelid family possessing remarkable regenerative ability, which is not only the response after traumatic injury, but also a key step during the life cycle of several syllid taxa. In these animals the posterior part of the body becomes an epitoke and is later detached as a distinct unit named stolon. Such a sexual reproductive mode is named schizogamy or stolonization. The prostomium and the proventricle, a modified foregut structure, have been proposed to have a control function during this process, though the concrete mechanisms behind it have never been elucidated.

**Results:**

By using different experimental set-ups, histology and immunohistochemistry combined with subsequent cLSM analyzes, we investigate and document the regeneration and stolonization in specimens of *Typosyllis antoni* that were amputated at different levels throughout the antero-posterior body axis. The removal of the anterior end including the proventricle implies an incomplete anterior regeneration as well as severe deviations from the usual reproductive pattern, i.e. accelerated stolonization, masculinization and the occurrence of aberrant stolons. The detailed anatomy of aberrant stolons is described. A histological study of the proventricle revealed no signs of glandular or secretory structures. The ventricle and the caeca are composed of glandular tissue but they are not involved in the reproductive and regenerative processes.

**Conclusions:**

As in other investigated syllids, the proventricle region has a significant role during stolonization and reproduction processes in *Typosyllis antoni*. When the proventricle region is absent, anterior and posterior regeneration are considerably deviated from the general patterns. However, proventricle ultrastructure does not show any glandular component, thereby questioning a direct involvement of this organ itself in the control of reproduction and regeneration. Our findings offer a comprehensive starting point for further studies of regeneration and reproductive control in syllids as well as annelids in general.

**Electronic supplementary material:**

The online version of this article (doi:10.1186/s12862-016-0770-5) contains supplementary material, which is available to authorized users.

## Background

Annelids provide excellent model organisms for investigating regenerative processes. Their regenerative capabilities are remarkable. They are able to renew worn structures such as chaetae or opercula [1, 2]. Furthermore, some annelids redevelop a complete body originated only in one or a few segments [3–5]. Studies concerning developmental biology in annelids and their regeneration patterns have been possible because the life-cycles of some of them are well-known and have been reproduced successfully under laboratory conditions. Such is the case of species of the clitellates *Enchytraeus*, *Helobdella* and *Pristina*, and polychaetes *Alitta, Capitella*, *Hydroides*, *Platynereis*, among others [6–15].

**Fig. 1:**
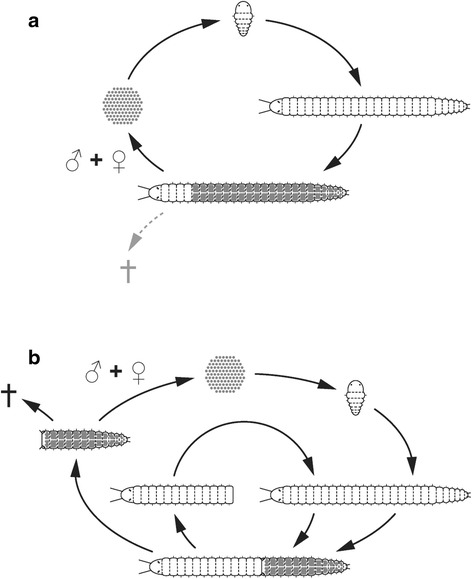
Special modes of sexual reproduction in annelids. **a** Epigamy. The specimen becomes an epitoke and produces gametes. After mating, the individual often dies. **b** Schizogamy. Only the posterior part of the individual is transformed into a separate epitokous unit called stolon. Gametes could be also produced in non-stolon segments and later transferred into the latter. After maturation, the stolon detaches, mates and dies, whereas the stock regenerates its posterior end

However, this group of model organisms represents very few of the numerous annelid lineages. Recently, members of other groups such as Syllidae, have been proposed as possible model species [16]. Syllids are interesting for developmental studies due to their complex life cycles and reproductive modes, named epigamy and schizogamy or stolonization, the latter include steps of segment regeneration [17–19]. During reproduction in several annelid families, the complete body is transformed into an epitoke, a process called epigamy. In contrast, during schizogamy only the posterior part becomes an epitoke, which is later detached as a distinct reproductive unit called stolon, while the posterior end of the stock is regenerated (Fig. 1).

The regeneration patterns of syllids and their reproductive methods were studied during the 20th century by Abeloos [20], Albert [21], Allen [22, 23], Berrill [24], Boilly [25–32], Boilly & Thibaut [33], Caullery [34], Deyle [35], Durchon [36–38], Durchon & Wissocq [39], Franke [18, 40–45], Gidholm [46], Hauenschild [47], Heacox & Schroeder [48], Izuka [49], Langhammer [50], Malaquin [51], Mesnil [52], Mesnil & Caullery [53], Michel [54], Müller & Kreischer [55], Okada [56–62], Pruvot [63], Schiedges [64], Verger-Bocquet [65–67], and Wissocq [68–73]. However, these studies did not continue during recent decades, and therefore new microscopic techniques could not be used. Some of the most relevant results suggested an endocrine function of the proventricle (a specific structure in the digestive tube) during the cyclic reproductive activity of *Typosyllis spp.* [38, 40–42, 44, 48, 68].

The proventricle is a muscular structure with radially arranged striated muscle cells surrounding the gut [74–77]. These cells consist of usually only one or two sarcomeres with up to 100 μm length, being the longest known sarcomeres within the Metazoa [78, 79]. Within the muscular fibers there are membrane-bound granules containing high amounts of calcium and phosphorus, contributing to the calcium metabolism of the muscle cells [79]. Several authors [38, 40, 69] proposed that the proventricle produces a hormone that, in high concentrations, attenuates the stolonization while promoting the regeneration of the posterior end. In later studies, Franke [41, 42, 44] as well as Heacox & Schroeder [48] suggested the prostomium as the main control core that manages the proventricle endocrine activity. However, these studies did not specify the hormone’s nature and the exact location for its production. Recently, Aguado et al. [16] suggested that the hormones might be produced by adjacent digestive structures that follow the proventricle, the ventricle and caeca, which are supposed to have glandular functions. However, a detailed histological study of these structures has not been performed and the specific control mechanisms of stolonization and regeneration continue to be unknown.

In this study, we investigate the anterior and posterior regeneration and the removal effect of the proventricle region in *Typosyllis antoni* (Syllinae) involving recent microscopical techniques. We used a variety of experimental set-ups, histology and immunohistochemistry combined with subsequent confocal laser scanning microscopy (cLSM) in order to investigate the relationships between regeneration, reproduction and the proventricle function.

## Methods

### Specimens and experimental setup

**Fig. 2:**
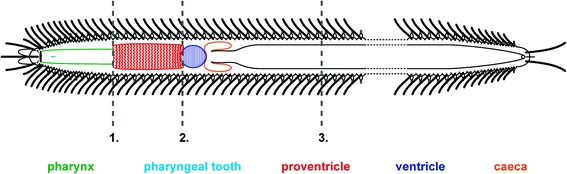
Schematic drawing of *Typosyllis antoni* illustrating the different cutting sites for each regeneration experiments: 1. In front of the proventricle, 2. Between proventricle and ventricle, 3. Between chaetigers 35 and 36

Specimens of *Typosyllis antoni* used in this study were taken from aquaria located at the University of Leipzig (Germany). In total, three different amputation sites were used in the regeneration experiments (Fig. 2): (1) amputation directly in front of the proventricle, (2) amputation at the border between proventricle and ventricle, and (3) amputation between chaetigers 35 and 36. Regeneration experiments 1 and 2 were performed during August and September 2014 with 18 specimens each. The specimens were anesthetized in 3.5 % MgCl_2_ dissolved in artificial seawater and amputated with a scalpel. Afterwards, anterior and posterior ends were placed separately in plastic bowls and kept at 25 °C with water changes every third day. Each experiment (amputation sites 1 and 2, respectively) was split into two sets with a delay of two days (set 1: days 2, 8, 10, 16; set 2: days 4, 6, 12, 14, 18). For each mentioned day, two specimens were removed, anesthetized and fixed in 4 % paraformaldehyde. An additional box was set up with three untreated individuals as a control. For more detailed information on animal maintenance please check reference [16]. A comparable setup for amputation in site 3 was conducted also in August and September 2014. This third experiment was performed to study the regenerating body ends during a longer time in order to check their ability to produce stolons. Two sets with six specimens each (numbers 1–1 to 1–6 and 2–1 to 2–6, respectively) were set up. In this experiment every specimen (anterior and posterior end) was placed in a distinct plastic bowl with the same conditions as described above. Over a period of 25 days (specimens 1–1 to 1–6) and 23 days (specimens 2–1 to 2–6) specimens were examined every day. Therefore, anterior and posterior ends were removed from their boxes, anesthetized in 3.5 % MgCl_2_ for 5–10 min, the number of segments was counted and modifications (e.g., stolon development) were documented. Afterwards, specimens were placed back in their box.

### Histology

For histology, specimens were treated as described previously [80], with the following modifications. Specimens were anesthetized in 7 % MgCl_2_ dissolved in artificial seawater, fixed overnight in Bouin's fixative (saturated aqueous picric acid, 37 % formaldehyde, glacial acetic acid; 15:5:1 by volume), washed in 70 % ethanol and dehydrated in an ascending ethanol series. Azan stains the neuropil of the nervous system gray, the extracellular matrix blue, and the musculature orange. The color of nuclei is variable based on their content.

### Immunohistochemistry

Immunohistochemistry was performed as described previously [5]. For this study, we used combined stainings of anti-α-tubulin or anti-serotonin with phalloidin-rhodamine. For anti-α-tubulin staining, a mixture of anti-acetylated α-tubulin (monoclonal anti-tubulin, acetylated antibody, produced in mouse, ascites fluid, Sigma-Aldrich, St. Louis, MO, USA; dilution 1:500 in PBST-NGS) and anti-tyrosinated α-tubulin (monoclonal anti-tubulin, tyrosine antibody, produced in mouse, ascites fluid, Sigma-Aldrich, St. Louis, MO, USA; dilution 1:250 in PBST-NGS) was used. The labeled α-tubulin is a structural component of microtubules, which are amongst others present in axons, while serotonin (=5-HT) is a neurotransmitter. Phalloidin-rhodamine labels filamentous muscular actin (f-actin) [5].

### Neutral red staining, light microscopy and image processing

For neutral red staining, a specimen was placed for 2 min in neutral red solution (2 mg/ml in artificial seawater) and afterwards anesthetized in 3.5 % MgCl_2_ dissolved in artificial seawater. Light microscopic pictures of neutral red staining and regenerating specimens were taken using a Leica (Leica Microsystems, Wetzlar, Germany) DM1000 microscope with attached Leica DFC295 camera and the Leica LAS v3.6 software. All drawings, images and final image plates were processed and compiled using Adobe (San Jose, CA, USA) Photoshop CS6 and Illustrator CS6.

## Results

### Proventricle morphology and function

**Fig. 3:**
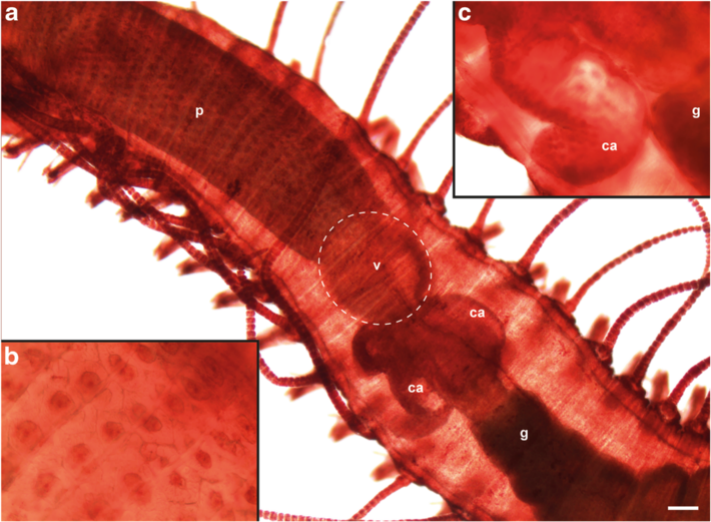
Light microscopic images of *Typosyllis antoni*, neutral red staining. Anterior end upper left, all images are dorsal views. **a** Modified foregut with proventricle (p), ventricle (v) and caeca (ca). **b** Higher magnification of proventricle surface. **c** Higher magnification of left caecum (c). Abbreviations: ca, caecum; g, gut; p, proventricle; v, ventricle. Scale bar = 100 μm

**Fig. 4:**
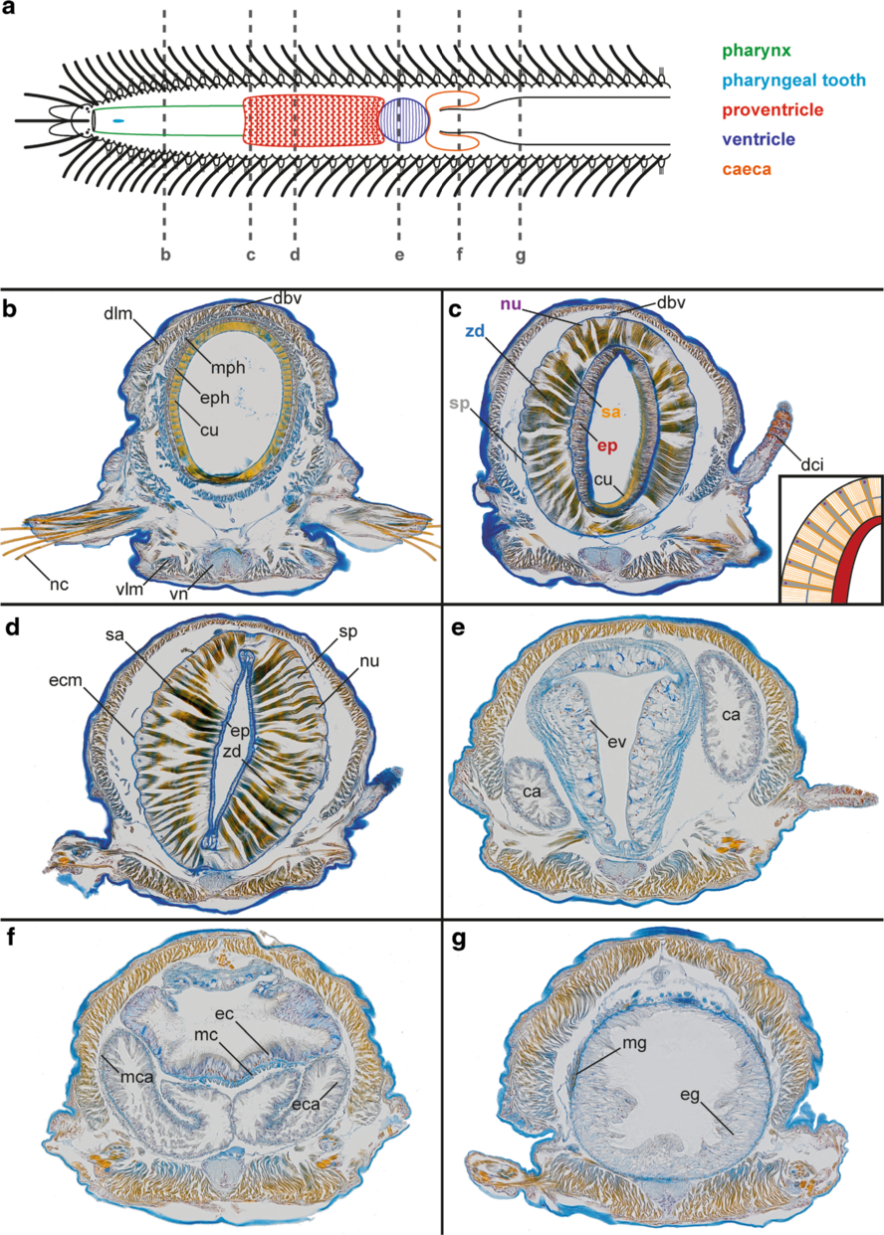
Histological cross sections of anterior segments in *Typosyllis antoni* (two different specimens, b-d respectively e-g), Azan staining. **a** Schematic representation of *T. antoni* with the locations of each cross section. **b** Pharynx. **c** Anterior end of proventricle and schematic representation of the upper left quarter of the proventricle (down right, with colors according to label colors). **d** Proventricle. **e** Ventricle. **f** Caeca region. **g** Midgut. Abbreviations: ca, caeca; cu, pharyngeal cuticle; dbv, dorsal blood vessel; dci, dorsal cirrus; dlm, dorsal longitudinal musculature; ec, gut epithelium of the caeca region; eca, caeca epithelium; ecm, extracellular matrix; eg, midgut epithelium; ep, epithelium of the proventricle; eph, pharyngeal epithelium; ev, epithelium of the ventricle; mc, gut musculature of the caeca region; mca, musculature of caeca; mg, midgut musculature; mph, pharyngeal musculature; nc, neurochaetae; nu, nucleus; sa, sarcomere; sp, sarcoplasm; vlm, ventral longitudinal musculature; vn, ventral nerve cord; zd, z-disc

The foregut of *Typosyllis antoni* is composed of a pharynx armed with one anterior pharyngeal tooth, followed by the proventricle, the ventricle and two caeca (Figs. 3a, 4a). The pharynx consists of an outer muscular layer, followed by a thin epithelium, which is covered by a prominent cuticle (Fig. 4b). At the transition from the pharynx to the proventricle, the cuticle becomes weaker, the epithelium is broadened and the lumen decreases (Fig. 4c). In contrast, the muscle layer enlarges, starting to show the typical radial arrangement of the sarcomeres (Fig. 3b). These sarcomeres are extremely long, thus only two sarcomeres per radius occur, as visible by the z-discs (connection site of actin filaments), on half cross-section of the proventricle musculature (Fig. 4c, d). The sarcoplasm is limited to tapered areas between the sarcomeres; the nuclei are present inside these areas, near the outer margin. Throughout the posterior end of the proventricle, the inner epithelium becomes gradually thinner, the musculature broadens and the interior lumen diminishes (Fig. 4d). Possible secretory structures are certainly not detectable within the proventricle. Instead, the ventricle has an abundance of glandular epithelium provided by cells with large vacuoles, while it almost lacks musculature (Fig. 4e). In the caeca region, the musculature starts to increase again and the large glandular cells get scarcer and smaller in size (Fig. 4f). The two caeca show an arrangement of tissues comparable to the following midgut (Fig. 4g), with a voluminous epithelium and a thin outer muscle layer (Fig. 4f, g).

### Regeneration differs across amputation sites

In order to investigate if anterior regeneration varies depending on the presence of the proventricle region and to exclude an involvement of the subsequent structures (ventricle + caeca), we tested three cutting sites (1, 2 and 3). Amputation site 1 (Fig. 2) was located directly anterior to the proventricle, so that the posterior body part kept the latter feature, but no additional parts of the pharynx. Amputation site 2 (Fig. 2) was at the border between the proventricle and the ventricle plus caeca, thus the posterior part lacks the proventricle, but kept the other structures. Amputation site 3 (Fig. 2) was between chaetiger 35 and 36, clearly behind the modified foregut.

**Fig. 5:**
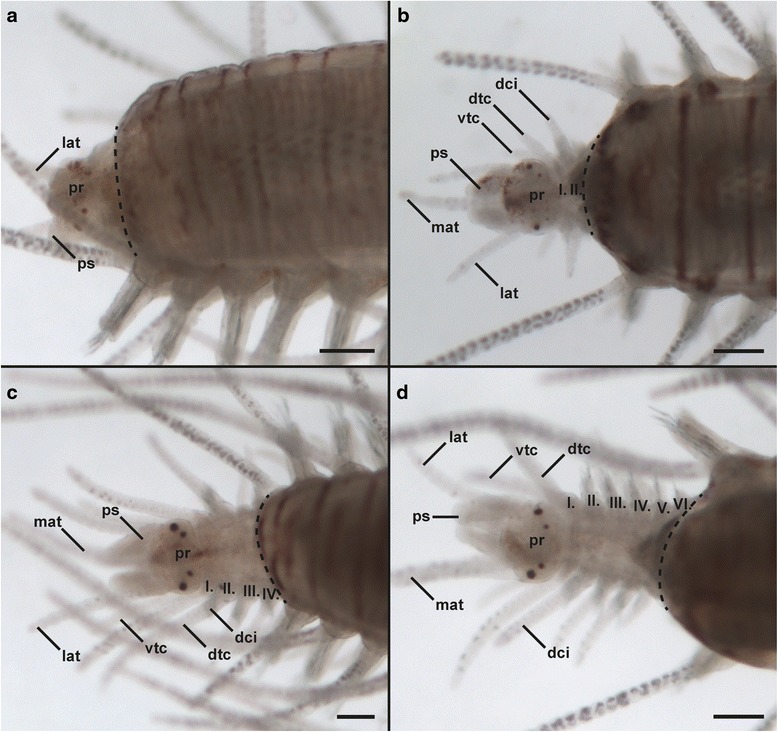
Anterior regeneration in *Typosyllis antoni* specimens amputated directly in front of the proventricle (Fig. 2, site 1), light microscopic pictures. All images are dorsal views of the anterior end except a (dorso-lateral), anterior is left in all images. The dotted line indicates the site of amputation. **a** 4 dpa. The prostomium (pr) and its appendages (lat, ps) are redeveloping but segments are not visible. **b** 10 dpa. Two more segments (I.-II.) have developed. **c** 12 dpa and **d** 14 dpa. Segments (III.-VI.) were added sequentially directly anterior to the amputation site. Abbreviations: I.-VI., regenerated segments; dci, dosal cirrus; dtc, dorsal tentacular cirrus; mat, median antenna; lat, lateral antenna; pr, prostomium; ps, palpus; vtc, ventral tentacular cirrus. Scale bars = 100 μm

*Amputation site 1 - anterior regeneration:* When amputating specimens directly in front of the proventricle, anterior regeneration starts with invagination followed by blastema formation and blastema patterning. This leads to the development of the prostomium with antenna and palps after 4 days post amputation (dpa) (Fig. 5a). Afterwards, new segments are regenerated consecutively directly anterior to the amputation site (re-segmentation) (Fig. 5b-d). The maximum number of observed segments was six after two weeks (Fig. 5d). Regeneration speed, represented by the number of new segments, varies between different specimens. All the specimens regenerated a pharynx but a new pharyngeal tooth was never observed.

*Amputation site 1 - posterior regeneration.* The anterior ends of specimens dissected in front of the proventricle regenerate slowly and are incomplete over the period of observation: After 14 dpa, about one third of the anterior body ends remained in the invagination stage without any signs of posterior regeneration (e.g., blastema formation). The remaining ones regenerated some few segments, but less than specimens amputated at sites 2 or 3. The maximum number of regenerated segments was four, first seen in a specimen at ten dpa.

*Amputation site 2 - anterior regeneration:* The anterior regeneration in specimens amputated between proventricle and ventricle bears striking differences in comparison with the other two sites. At first, two or usually three segments were visible at once (first seen 6 dpa) and no more segments were added until 16 dpa. At 18 dpa, one specimen showed in total four and the other one five regenerated segments. Thus, additional segments were added sequentially.

*Amputation site 2 - posterior regeneration:* Posterior regeneration in specimens amputated between proventricle and ventricle is faster than in site 1. The maximum number of regenerated segments observed was 16 in a specimen at 18 dpa. However, not all the individuals regenerated at the same rate and some remained in the invagination stage for more than one week without any signs of blastema formation. In addition, during the experiment, a redevelopment of the ventricle and caeca was not observable.

*Amputation site 3 - anterior regeneration:* In this case, *T. antoni* usually regenerates two or sometimes three segments visible at once at 4 dpa. Afterwards the prostomium and the segments grow, but no more segments were added within one month after (Table 1, for more details and pictures please refer to [16]).

**Table 1:** Summary of regeneration in *Typosyllis antoni* after dissecting at site 2 and 3

dpa	regeneration
*amputation site 2 – anterior regeneration (observation only every 2* ^*nd*^ *day)*	
0	invagination of 1st remaining segment at wound site
2	blastema formation
4	prostomium with developing antenna, palps and eyes
6–16	two or three segments
18	sequential addition of further segments
*amputations site 2 – posterior regeneration (observation only every 2* ^*nd*^ *day)*	
0 + 2	invagination of 1st remaining segment at wound site
4	pygidium with developing anal cirri and median papilla
6 onwards	sequential addition of segments
*amputation site 3 – anterior regeneration*	
0–1	invagination of 1st remaining segment at wound site
2	blastema formation
3	blastema well-developed
4	prostomium with developing antenna, palps and eyes; two or three segments with tentacular cirri regenerating in 1st segment
6	pharynx present
7 onwards	growth of segments and structures
*amputation site 3 – posterior regeneration*	
0–2	invagination of 1st remaining segment at wound site
3	blastema well-developed
4	pygidium with developing anal cirri and median papilla
5	addition of first new segment
6 onwards	addition of further new segments followed by their development

*Amputation site 3 - posterior regeneration:* The posterior regeneration in specimens amputated between chaetiger 35 and 36 is comparable to amputation site 2.

### Stolonization and stolon morphology

**Fig. 6:**
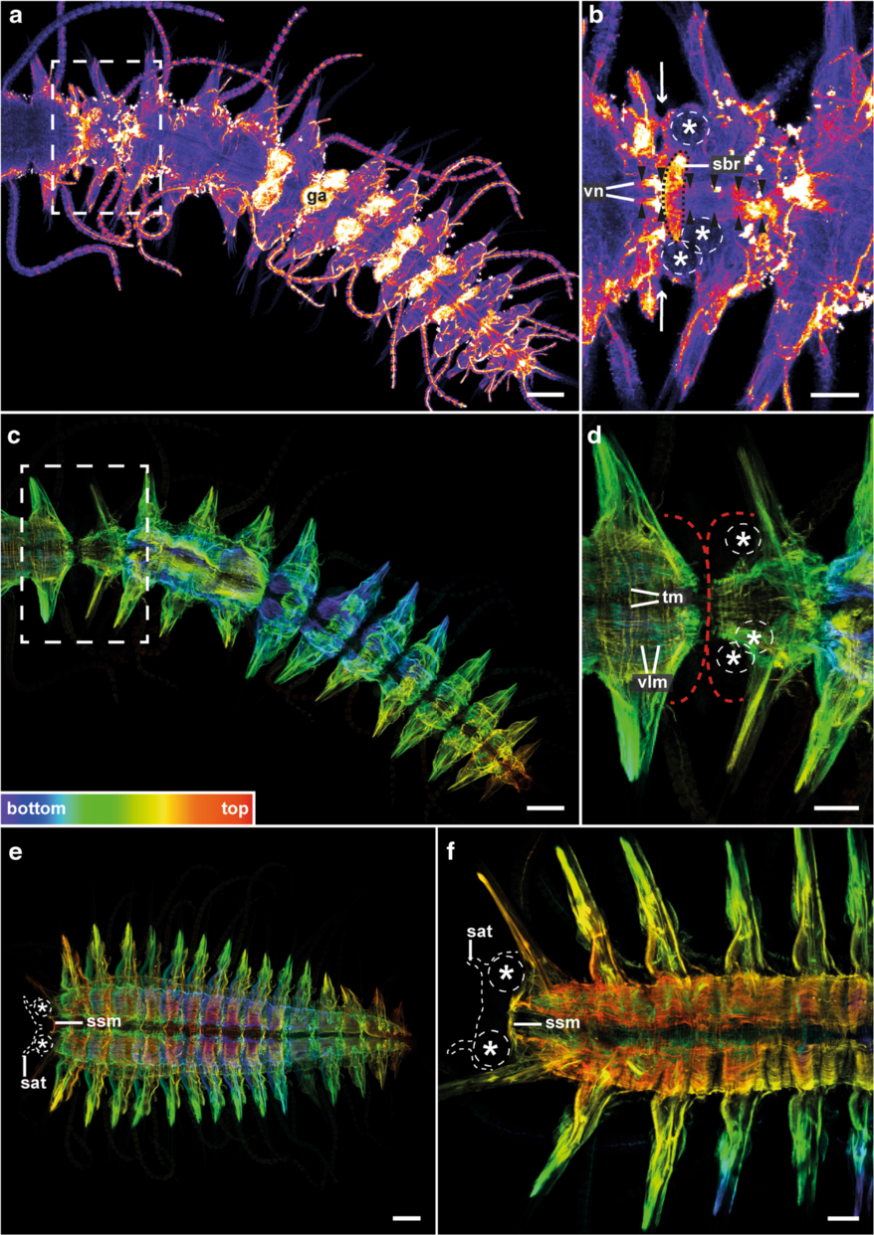
Attached (**a**-**d**) and detached (**e**-**f**) stolons of *Typosyllis antoni*. Anti-α-tubulin (glow mode, a-b) and anti-f-actin (depth coded, legend inserted in c, c-f) staining, confocal maximum projections. Anterior is left, all images are ventral views. Arrows in b indicate the border between stock and stolon, asterisks designate eyes, red dotted lines in d indicate edges of stock (anterior) and attached stolon (posterior) while white dotted lines in e and f indicate the anterior margin of the stolon. **a** Overview and **b** detailed image of marked area with reduced stack number. Near the detachment site and between the eyes, a dorsal orientated loop coming from the ventral nerve cord appeared, representing the stolon brain (sbr). The ventral nerve cords (vn; arrowheads) of stock and stolon were still connected. **c** Overview and **d** detailed image of marked area. The musculature is only weakly developed at the transition between the remaining body and the stolon. At the anterior margin of the stolon, the transverse musculature (tm) appears contracted. **e** Male stolon and **f** stolon of unknown sex. In front of a level between the parapodia of the stolon head, no musculature is visible. At this level the transverse musculature formed a sphincter muscle (ssm). Abbreviations: ga, gamete; sat, stolon antenna; sbr, stolon brain; ssm, stolon sphincter muscle; tm, transverse musculature; vlm, ventral longitudinal musculature; vn, ventral nerve cord. Scale bars = 100 μm (**a**, **c**, **e**), 50 μm (**b**, **d**, **f**)

Stolons of *T. antoni* are dicerous, possessing two anterior lobes, two pairs of eyes and two anterior antenna [16]. While female stolons are full of gray oocytes, males contain two packages of yellow sperm per segment. *T. antoni* can pass through several successive stolonization events. Stolons in *T. antoni* are not generated by segment addition, but arise from a transformation of the posterior segments. After detachment of a stolon, the posterior end is regenerated before new stolons are developed. The number of transformed segments varies between specimens, and presumably also between each stolonization event in a single specimen, from about 5 to 18 segments. The anteriormost stolon segment bearing the eyes and antenna underwent extensive morphological changes and became the stolon “head”. Additionally, the nervous system and the musculature passed through a remodeling process. Already in the still attached stolon, a dorsal orientated ring of neurites occurred at the anterior margin of the stolon head (Fig. 6a, b). Having its origin in the ventral nerve cord, this structure represents the stolon brain. Nevertheless, the ventral nerve cord was not interrupted between the remaining body and the stolon at this stage. Moreover, the body wall musculature is reduced at the border between the remaining body (stock) and the stolon (Fig. 6c, d). Inside the stolon head, the transverse muscle fibers contract to rings which later become a sphincter muscle prior to detachment (Fig. 6e, f). The anterior third of the stolon head is free of musculature. All further stolon segments exhibited a nervous system and musculature comparable to stock segments.

### The effect of prostomium and proventricle region removal during stolonization

In order to get a better understanding of potential relationships between regeneration and stolon development, 12 additional individuals were amputated between chaetiger 35 and 36 (site 3, Fig. 2). The anterior and posterior ends (latter ones without a proventricle) were monitored over a longer period to investigate the production of stolons.

*Posterior regeneration -* During posterior regeneration (anterior body parts), all 12 specimens showed an invagination at 1 dpa. One specimen was not found after 2 dpa and another specimen remained at this stage until the end of the experiment (25 dpa) without any signs of regeneration. The other ten regenerated their posterior ends, but in three specimens, after two weeks, the posteriormost part was lost and regenerated again. The maximum number of segments regenerated was 24 (without loss of segments; 25 dpa), the minimum 11 (23 dpa). In those specimens, which lost their posterior end, a total of 30 segments were regenerated. None of the examined specimens produced stolons. The lost posterior ends are certainly traumatic losses, as we did not observe gametes, attached stolons, or detached stolons in those specimens and their bowls.

**Fig. 7:**
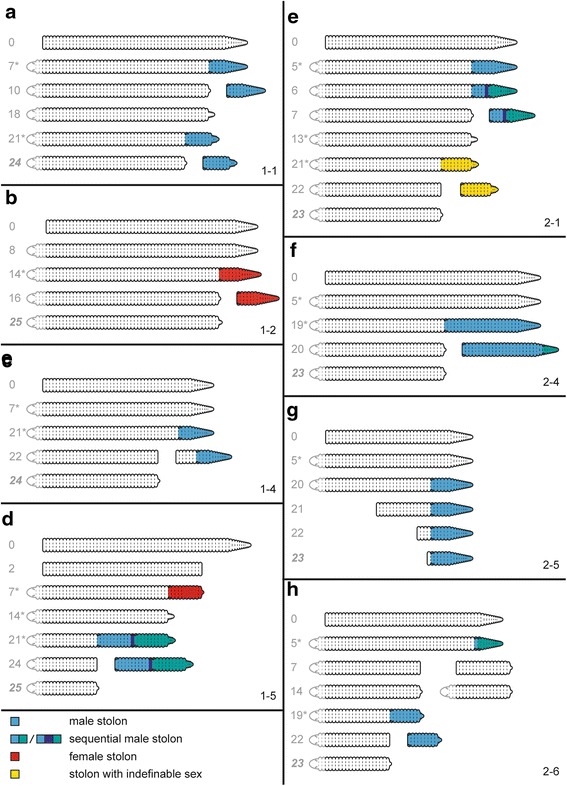
Schematic representation of eight selected *Typosyllis antoni* specimens monitored during anterior regeneration after amputation between chaetigers 35 and 36 (Fig. 2, site 3). Numbers in the lower right corner refer to each specimen. Grey numbers denote days post amputation, asterisks indicate that at least one day before the specimen was not observed. Last drawing of each specimen is always the last day of observation. **a** Specimen with subsequent development of two male stolons. **b** Specimen developing only one female stolon. **c** Individual with scission site some segments in front of the male stolon. **d** After developing a female stolon, this specimen developed an aberrant sequential stolon composed of three partially incomplete male stolons, which later detached as a whole. **e** The initially developed male stolon was transformed into an aberrant sequential one, the second stolon looked normal. **f** The developed male stolon was much longer than usually and later became an aberrant one composed by two subsequent stolons, which detached as a whole. **g** This specimen lost sequentially several anterior segments resulting in a male stolon with only one additional stock segment. **h** After developing an aberrant male stolon, this specimens broke in two parts, which both started regeneration of each correspondent missing body end. After that, the anterior body part developed a normal male stolon

*Anterior regeneration -* During anterior regeneration (posterior body parts without proventricle and prostomium) all 12 specimens regenerated a new prostomium and usually two new segments (Fig. 7, Additional file 1: Figure S1). Only in specimen number 1–2 (Fig. 7b) and a midbody fragment of specimen 2–3 (Additional file 1: Figure S1d) three regenerated segments were observed. All specimens developed stolons; seven specimens went through only one stolonization event, while five experienced two successive stolonizations. The detachment was produced several segments anterior to the stolon in specimens 1–4 and 2–5 (Fig. 7c, g), while in specimens 1–5, 2–6, and 2–3 (Fig. 7d, h, Additional file 1: Figure S1d) the anterior body was fragmented previously to the development of the stolon. The number of segments within each single stolon was between ten and 17 segments. Four specimens (1–5, 2–1, 2–4, 2–6, Fig. 7d, e, f, h) produced aberrant male stolons (described below) in series of two or three, which were detached as a whole. Only in three specimens (1–2, 1–5, 1–6, Fig. 7b, d, Additional file 1: Figure S1b) female stolons occurred. In some individuals (e.g., 2–1, Fig. 7e) the stolon was already developed within less than one week after amputation, in others (e.g., 1–4; Fig. 7c) this lasts nearly three weeks. During this time, the former went through their second stolonization event (e.g., 1–1, Fig. 7a). Neither the number of sequential stolons nor the relative position of the first stolon head within the aberrant stolons showed a regular pattern.

Accelerated stolonization, masculinisation and the occurrence of aberrant stolons has been also observed in the specimens amputated at site 2. In contrast, none of these changes was observable in specimens amputated at site 1.

### Morphology of aberrant stolons

**Fig. 8:**
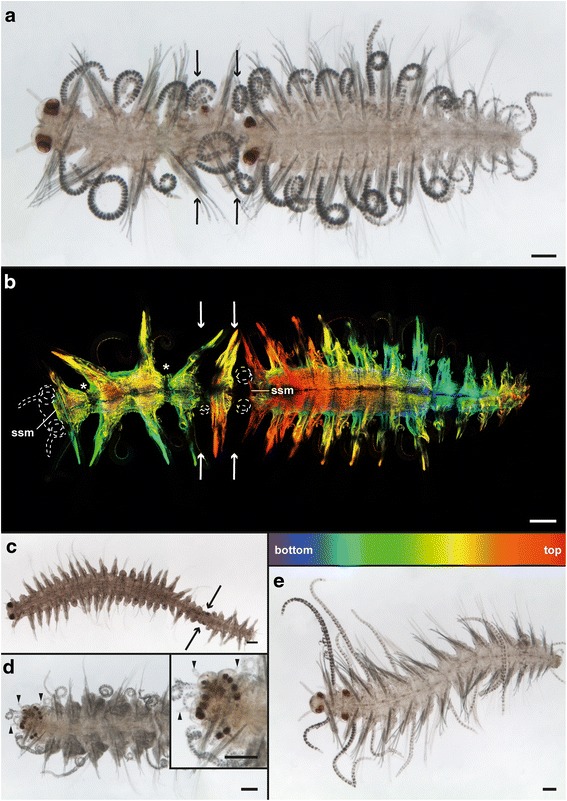
Aberrant stolons of *Typosyllis antoni*. Light microscopic images (a, c-e) and confocal maximum projection of anti-f-actin staining (b, depth coded, legend below image), anterior is left in all images. **a** Dorsal and **b** ventral view of a sequential stolon showing two complete and one imperfect head. Borders between the distinct stolons are indicated by arrows, dotted lines in b indicate anterior margin and eyes. The asterisks highlight two segmental borders in the anteriormost stolon, which also showed signs of muscle degeneration. **c** Sequential stolon with a longer anterior one and a poorly developed second one. Arrows indicate borders between the two stolons. **d** Aberrant stolon with anterior end showing a fusion of heads, as visible in the increase in eye number (insert with close-up). Arrowheads indicate the antenna. **e** Sequential stolon composed of two stolons with the first one being only represented by one segment. Abbreviations: ssm, stolon sphincter muscle. Scale bars = 100 μm

Aberrant stolons showed a great variety of segment number and order (Fig. 8a, c-e). Some of them were composed of two (Fig. 8c, e), others of three (Fig. 8a) sequential stolons at different developmental stages. In one case, the anterior end appeared to be a composition of two segments with a total of 11 eyes, three antennae but only one parapodium (Fig. 8d). In this case, the stolon head was induced three times through two subsequent segments. The musculature of the aberrant stolons also shows deviations from the regular pattern (Fig. 8b). The sphincter muscles of the first and the third stolon are well developed, but there are few muscle fibers left between the stolon two and three, indicating that this stolonization event is younger. This is especially true for the second stolon, indicating that this stolonization initiation is the youngest. Additionally, within the first stolon, musculature starts fading at two segmental borders (Fig. 8b, asterisks), maybe representing the initiation of two more stolon heads.

## Discussion

Removal of the proventricle region has strong effects on the processes of stolonization and posterior regeneration. Our results are in agreement with previous studies [37, 40, 48, 69] and clearly support an additional regulatory function of this region. The presence of a proventricle has been proposed as a synapomorphy for Syllidae, and its ancestral function is seen in the role of a sucking pump [81, 82]. Comparable structures have not been found in the digestive tract of any other group of annelids [83]. The proposed sucking function may be particularly useful in many syllids that feed on hydroids, bryozoans or other invertebrates [75, 84]. However, many other syllids show a different kind of feeding preferences [85–87], e.g., algae as in the *T. antoni* diet. These different feeding strategies are widely spread especially within Syllinae, where the individuals reproduce by schizogamy. Thus, the proventricle might have been coopted from a plesiomorphic sucking pump function into an additional stolonization control purpose. This might explain its presence in a group of annelids that show a high diversity in reproductive modes, but do not seem to primarily rely on it for feeding [17, 81]. Alternatively, structures located in the proventricle region (e.g., nervous system) might be responsible for the regulation of reproductive and regenerative processes.

**Fig. 9:**
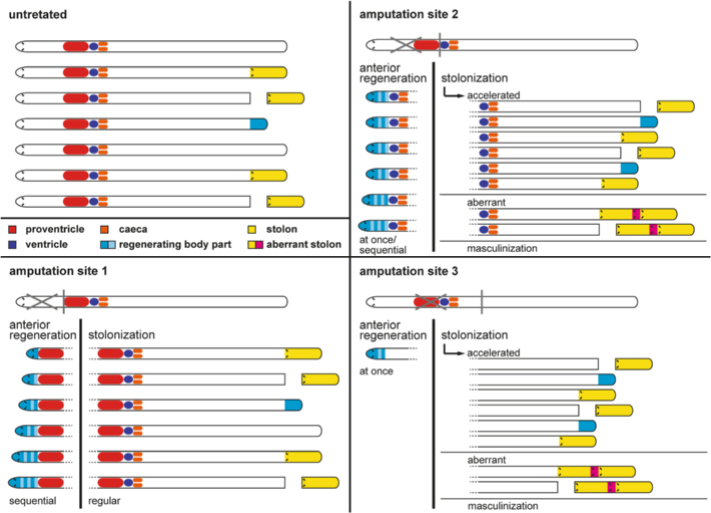
Schematic comparison of amputation site effects, anterior and posterior regeneration and stolonization

According to our results, the effects of the removal of the proventricle region in the stolonization-posterior regeneration processes are: 1) The stolonization is accelerated; 2) only three segments and the pygidium are regenerated after stolonization; 3) usually only male stolons are produced; and 4) appearance of aberrant stolons (Fig. 9). H-D Franke [40] also found that the removal of the proventricle caused acceleration in the production of stolons in *S. prolifera*. In this species, only 3–4 posterior segments plus the pygidium are regenerated after proventricle removal [40]. An accelerated stolonization also occurs in proventricle-less *T. pulchra* but to a minor degree [48]. Additionally, in *S. prolifera* as well as in *T. pulchra* the male sex of stolons might be linked to the absence of the proventricle signalling, which seems necessary for female determination [40, 48]. In our results, a few female stolons (specimens 1–2, 1–5, and 1–6; Fig. 7, Additional file 1: Figure S1) occur in the first stolonization; however the sex of these stolons had probably been determined prior to amputation. Aberrant stolons were also found in the regenerating *Procerastea halleziana*, a member of Autolytinae, after the amputation of the proventricle [50].

Therefore, a relationship between the proventricle and stolonization-regeneration can be assumed. However, the specific way the proventricle controls these processes is not clearly established. Franke [41, 42] proposed an endocrine function of the proventricle itself, controlled by the prostomium. Two factors might be involved: the "stolonization-inhibiting hormone" released by the proventricle; and the "stolonization-promoting hormone" mediated by the prostomium. Additionally, the proventricle hormone would not be exclusively inhibiting stolonization, but a low concentration might be also necessary for timing reproductive processes. As well, the prostomial signal might not only target the proventricle, but also other structures involved in reproduction [41]. A major reproductive control function originating in the prostomium is also described in other Phyllodocida such as *Eulalia viridis*, *Harmothoe imbricata*, or *Hediste diversicolor* [88–90]. A recent study on Nereidae revealed a high complexity of reproduction control, described by a multi-hormone model [91].

However, morphology does not support an endocrine function of the proventricle itself. The proventricle of syllids is basically a muscle structure. It is composed of large sarcomeres [74–76, 78, 92, 93] and granules with high amounts of phosphorus and especially calcium ions [79]. Our histological survey has revealed that there is no other feature associated in the proventricular region, and there are no signs of glandular tissue through the whole proventricular structure. However, the proventricle muscle cells contain large plasmatic areas and remarkably huge nuclei (Fig. 4c, d), suggesting a high metabolic activity.

It has been suggested that the muscle cells of the proventricle in *Syllis spongiphilia* have a myoepithelial origin [76–78]. Myoepithelial cells have been described with secretory functions in other groups of organisms [94]. However, our results and that of other previous authors contradict this possible myoepithelial origin. The muscle cells have no epithelial part and are demarcated with an extracellular matrix (presumably basal lamina) on both sides, close to the coelom as well as to the epithelium that surrounds the gut lumen (Fig. 4d). Additionally, the presence of an inner and an outer epithelium encircling the proventricle musculature in *Syllis gracilis* has been demonstrated using transmission electron microscopy [33]. Obviously, the use of the term “myoepithelial” was either a misinterpretation or a misuse. The proventricle muscle cells are characteristically striated muscle cells with no secretory function.

The proventricle muscle cells contain high amounts of calcium ions. Recently, it has been demonstrated that intracellular calcium ion signalling is essential in the regulation of cell activity in intestinal stem cells of *Drosophila* [95]. However, it is likely that the calcium in the syllid proventricle is related to the muscular function as well, in particular because extracellular calcium ion signalling has not been found [79, 95].

On the other hand, in *Myrianida prolifera* the stolonization process seems to be normal in females when only the proventricle is extirpated, not the complete proventricle region [47]. However, the proventricle in *Typosyllis* spp., has repeatedly shown to be the control core through successive extirpation and reimplantation experiments, both in male and female specimens [37, 40–42, 48]. Additionally, the histological analyses performed herein clearly reveal that proventricular segments do not show any structural difference to other segments, apart from the proventricle itself.

In a previous study we suggested that the ventricle and caeca might be responsible for the endocrine control role during stolonization [16]. Indeed, our histological sections clearly show glandular and secretory tissues in these structures (Fig. 4e-g). However, specimens amputated between the proventricle and the ventricle (Fig. 2, amputation site 2) show the same differences in stolonization as those amputated far behind the complete modified foregut (Fig. 2, amputation site 3). This suggests that the ventricle and the caeca have no influence on the stolonization and thus, only a digestive function seems to be likely for these structures. Considering all of the evidence together, the exact role of the proventricle during reproduction and regeneration still remains obscure.

Our results further suggest that the removal of the proventricle region is influencing the anterior regeneration. When the proventricle region is present in the posterior end, the anterior regeneration seems to be a gradual process with the possible participation of an anterior segment addition zone. A similar pattern has been reported for *Branchiomma luctusosum*, *Dorvillea bermudensis* or *Syllis gracilis* [33, 96, 97]. However, when the proventricle region is removed, the regeneration process shows deviations from the general pattern. In this case the first segments appear simultaneously. Such a pattern of regeneration has also been found in *Cirratulus* cf. *cirratus*, *Enchytraeus japonensis*, *Euyrthoe complanata, Cirrineris* sp. or *Timarete* cf. *punctata,* amongst others [5, 98–102]. There are two different explanations for the simultaneous development of segments after the removal of the proventricle region. Firstly, the segments may have been determined one after another by an anterior segment addition zone, but too fast to observe a difference in timing. Secondly, the regeneration process might have begun with the redevelopment of tissue followed by seperation into different segments. In the latter case, the presence of a segment addition zone is not necessary and segments are formed simultaneously. In any case, the proventricle region seems to play a control function not only in posterior regeneration and stolonization, but also influences anterior regeneration.

## Conclusion

The syllid proventricle is a structure composed of striated muscle cells with giant sarcomeres and prominent calcium concretions, but without any signs of putative glandular tissue. All experimental data so far clearly suggests that the proventricle (region) influences regenerative and reproductive processes of these animals. Effects of proventricle region removal include stolonization acceleration, limited posterior segment regeneration after stolonization, masculinization, and appearance of aberrant stolons (Fig. 9). Proventricle region removal also seems to influence anterior regeneration, which either could be mediated by a segment addition zone or simultaneous formation of segments. Future experiments including dissection and regrafting of the proventricle, repeated amputation of the prostomium, as well as gene expression studies will allow to establish the robustness of our results and will provide a comprehensive understanding of syllid stolonization, regeneration, and their relationship.

## Additional file

Additional file 1: Figure S1. Schematic representation of four *T. antoni*. specimens monitored during anterior regeneration after amputation between chaetigers 35 and 36 (amputation site 3). Numbers in the lower right corner refer to the specimen. Grey numbers denote days post amputation, asterisks indicate that at least one day before was not observed. Last drawing of each specimen is always the last day of observation. a Specimen with subsequent development of two male stolons. b Specimen with development of only one female stolon. c Specimen with development of only one male stolon. d Specimen with development of only one male stolon. After stolon detachment, the remaining body broke in parts and only a midbody fragment regenerating its anterior end was found. (TIF 1341 kb)
