# Supplementary figures and images for: On the role of the proventricle region in reproduction and regeneration in Typosyllis antoni (Annelida: Syllidae)
Source: BMC Evol Biol. 2016 Oct 4;16:196. doi: 10.1186/s12862-016-0770-5 (PMC5050598; doi:10.1186/s12862-016-0770-5)

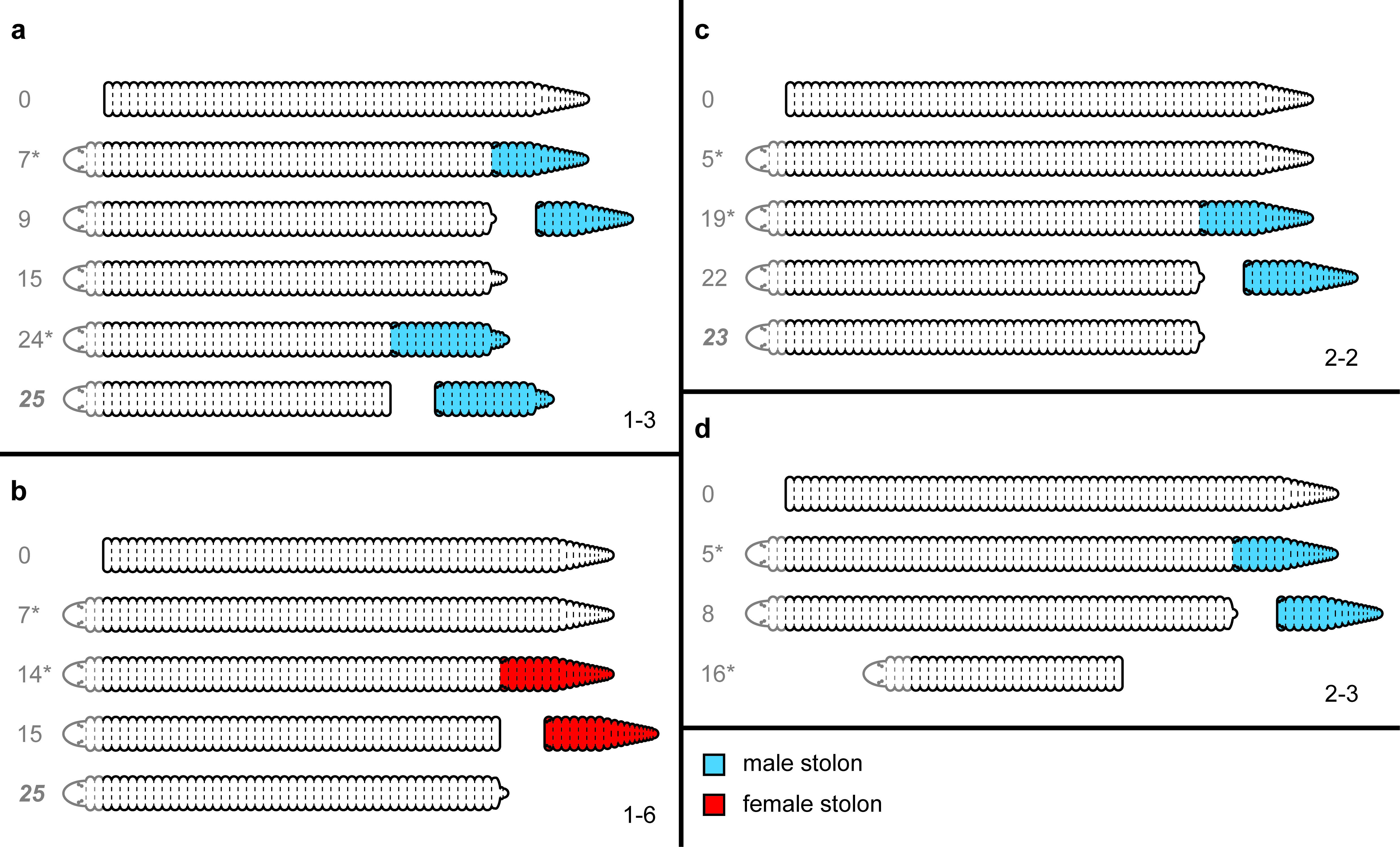

Supplement: Additional file 1: Figure S1. — Schematic representation of four T. antoni. specimens monitored during anterior regeneration after amputation between chaetigers 35 and 36 (amputation site 3). Numbers in the lower right corner refer to the specimen. Grey numbers denote days post amputation, asterisks indicate that at least one day before was not observed. Last drawing of each specimen is always the last day of observation. a Specimen with subsequent development of two male stolons. b Specimen with development of only one female stolon. c Specimen with development of only one male stolon. d Specimen with development of only one male stolon. After stolon detachment, the remaining body broke in parts and only a midbody fragment regenerating its anterior end was found. (TIF 1341 kb) [file 12862_2016_770_MOESM1_ESM.tif]
